# Supplementary material for: Content-rich biological network constructed by mining PubMed abstracts
Source: BMC Bioinformatics. 2004 Oct 8;5:147. doi: 10.1186/1471-2105-5-147 (PMC528731; doi:10.1186/1471-2105-5-147)
Supplement: Additional File 2 — The original results of the above study (non-essential files are deleted to keep the file size under the limit set by BMC bioinformatics). [file 1471-2105-5-147-S2.bz2 › chilibotAdditionalFile2/dip10/400ID8955093E9955/html/LIF_LIFR.html]

 


 **LIF** and **LIFR** 
  
Found 183 abstracts in PubMed, retrieved 10.  
 

 What does Google say? 
 PDF only 
| .edu only 

---

**Interactive relationship** (e.g. stimulation, inhibition, etc)

**Neutral relationship**- Previous studies suggested that the NH 2 terminal CBM and the Ig like domain of the  **LIFR**  are most important for  **LIF**  binding and activity.  Ref: 12731865 Biochemistry, 2003
- Our results indicate that  **LIF** , CT 1 and OSM share an overlapping binding site located in the Ig like domain of  **LIFR** .  Ref: 12707269 J Biol Chem< MedlineTA>J Biol Chem, 2003
- The different behavior of  **LIF**  and CT 1, on one side, and of OSM, on the other side, can be related to the different affinity of their site III for  **LIFR** .  Ref: 12707269 J Biol Chem< MedlineTA>J Biol Chem, 2003
- Signal transduction by CNTF requires that it bind first to CNTF R, permitting the recruitment of gp130 and  **LIF**  R  [ **LIFR** ] , forming a tripartite receptor complex.  Ref: 12765290 J Zhejiang Univ Sci< MedlineTA>J Zhejiang Univ Sci, 2003

**Non-interactive relationship** (e.g. studied together, co-existance, homology, etc.)

- Here we report that the expression of leukemia inhibitory factor  [ **LIF** ]  receptor  **LIFR** , one of the signaling molecules shared by several neuropoietic cytokines of the IL 6 family, is specifically upregulated in PC12 cells following treatment with NGF.  Ref: 12871977 J Biol ChemJ Biol Chem, 2003
- Human CNTF elicits cellular responses by induction of a receptor complex consisting of the CNTF alpha receptor CNTFR, which is not involved in signal transduction, and the beta receptors gp130 and leukemia inhibitory factor  [ **LIF** ]  receptor  **LIFR** .  Ref: 12643274 J Biol Chem, 2003
- We concluded that the fusion protein Hyper CNTF had superagonistic activity on target cells expressing gp130 and  **LIF**  R  [ **LIFR** ] , but lacking membrane bound CNTF R.  Ref: 12765290 J Zhejiang Univ Sci< MedlineTA>J Zhejiang Univ Sci, 2003
- Leukemia inhibitory factor  [ **LIF** ]  receptor  **LIFR** , epidermal growth factor receptor EGFR, and their respective ligands have been implicated in regulating growth and development of the early pig conceptus.  Ref: 12698007 Cytogenet Genome Res, 2002
- The functional receptor complex of ciliary neurotrophic factor CNTF, a member of the gp130 family of cytokines, is composed of CNTF, the CNTF receptor alpha CNTFR, gp130 and the leukemia inhibitory factor  [ **LIF** ]  receptor  **LIFR** .  Ref: 12707266 J Biol Chem< MedlineTA>J Biol Chem, 2002
- **LIF**  receptors, gp130 and  **LIFR**  alpha were significantly upregulated in cHI P &lt.  Ref: 12799551 AIDS< MedlineTA>AIDS, 2002
- Because corticosterone is known to have a broad range of effects and  **LIF**  function has been associated with the hypothalamo pituitary adrenal axis, this study was designed to determine the role for  **LIFR**  in the fetus when exposed to the elevated maternal glucocorticoid levels of late gestation.  Ref: 12823859 Reprod Biol Endocrinol< MedlineTA>Reprod Biol Endocrinol, 2002
- BACKGROUND Ablation of the low affinity receptor subunit for leukemia inhibitory factor  [ **LIF** ]   **LIFR**  causes multi systemic defects in the late gestation fetus.  Ref: 12823859 Reprod Biol Endocrinol< MedlineTA>Reprod Biol Endocrinol, 2002
- In contrast to other hematopoietic cytokine receptors, the leukemia inhibitory factor  [ **LIF** ]  receptor  **LIFR**  possesses two cytokine binding modules CBMs.  Ref: 12731865 Biochemistry, 2003
- Molecular modeling of the complex of  **LIF**  with the Ig like domain of  **LIFR**  provides a clue for the superadditivity of the D214A F284A double mutation.  Ref: 12707269 J Biol Chem< MedlineTA>J Biol Chem, 2003
- The proliferative results confirmed that CNTF required homodimerization of the gp130, CNTF R and  **LIF**  R  [ **LIFR** ]  receptor subunit whereas Hyper CNTF required heterodimerization of the gp130 and  **LIF**  R  [ **LIFR** ]  receptor subunit.  Ref: 12765290 J Zhejiang Univ Sci< MedlineTA>J Zhejiang Univ Sci, 2003
- BACKGROUNDLeukemia inhibitor factor  **LIF** , a member of the interleukin 6 cytokine family, has recently been shown to inhibit HIV 1 replication both in vivo and in vitro.OBJECTIVELIF and its corresponding receptors gp130 and  **LIF**  receptor alpha  **LIFR**  alpha were studied in lymphoid tissue LT to reveal potential systemic immunoregulatory effects during the course of HIV 1 infection.METHODSLIF, gp130,  **LIFR**  alpha and HIV 1 replicating cells were identified at the single cell level by immunohistochemistry and quantified by computerized in situ imaging in tonsil and lymph nodes biopsies LT from patients with primary HIV 1 infection PHI, chronic HIV 1 infection cHI, long term non progressors LTNP and HIV 1 seronegative controls.RESULTSLIF and its receptors, gp130 and  **LIFR**  alpha were significantly P &lt.  Ref: 12799551 AIDS< MedlineTA>AIDS, 2003
- Mapping and microsatellite marker development for the porcine leukemia inhibitory factor  [ **LIF** ]  receptor  **LIFR**  and epidermal growth factor receptor EGFR genes.  Ref: 12698007 Cytogenet Genome Res, 2002
- Cells that only express gp130 and  **LIF**  R  [ **LIFR** ] , but not CNTF R are refractory to stimulation by CNTF.  Ref: 12765290 J Zhejiang Univ Sci< MedlineTA>J Zhejiang Univ Sci, 2002
- The leukemia inhibitory factor  [ **LIF** ]   **LIF** , cardiotrophin 1 CT 1 and oncostatin M OSM are four helix bundle cytokines acting through a common heterodimeric receptor composed of gp130 and  **LIF**  receptor  **LIFR** .  Ref: 12707269 J Biol Chem< MedlineTA>J Biol Chem, 2002
